# Translation and validation of the Portuguese version of the “Care of the Dying Evaluation”

**DOI:** 10.1590/1806-9282.20250704

**Published:** 2026-03-30

**Authors:** Juliana Nalin Passarini, Vilma Adriana Tripodoro, Catriona Mayland, Dagny Faksvåg Haugen, Lair Zambon, Ivete Alonso Bredda Saad

**Affiliations:** 1 Universidade Estadual de Campinas, Faculty of Medical Sciences – Campinas (SP), Brazil.; 2 Instituto Pallium Latinoamérica – Buenos Aires, Agentina.; 3 The University of Sheffield – Sheffield, England.; 4 University of Bergen – Bergen, Norway

**Keywords:** Palliative care, Terminal care, Quality of death

## Abstract

Ensuring cancer patients and their families receive high-quality care at the very end of life is of paramount importance. The manner a family member dies has a profound impact on the bereaved. One way to assess this care is by using a post bereavement questionnaire.

**OBJECTIVE:**

The aim of the study was to translate into Brazilian Portuguese, conduct cross-cultural adaptation, validate and use a post-bereavement tool, “Care of the Dying Evaluation”.

**METHODS:**

An observational, international, cross-sectional multicenter study in seven countries, through 2018 to 2020. In Brazil, participants were recruited from two hospitals, Hospital Estadual Sumaré and Hospital das Clínicas, and had been admitted for a minimum of 3 days before death. Translation of CODE^TM^ in keeping with international principles, and pre-testing using patient and public involvement and cognitive interviews with bereaved relatives.

**RESULTS:**

The survey involved 235 bereaved family members, mostly female (70.6%), with 40% being sons and daughters of the deceased. Validity of the CODE^TM^ tool was assessed using confirmatory factor analysis, internal consistency through Cronbach’s α, and reliability via the Kappa coefficient and Spearman’s correlation. The study compared care quality perceptions between those who received palliative care and others. Findings revealed that 50.7% were informed about the dying process, and over 60% expressed that clarification of expected symptoms would have been beneficial.

**CONCLUSION:**

The study confirms that the CODE^TM^ questionnaire is a valid and reliable tool for evaluating the quality of end-of-life care within the Brazilian Portuguese context.

## INTRODUCTION

The World Health Organization (WHO) reports that approximately 58 million people die worldwide annually, with cancer being the second leading cause of mortality. In 2020 alone, cancer accounted for around 9.9 million deaths, many of which involved insufficient management of pain and other distressing symptoms^1^. In 2015, the Economist Intelligence Unit evaluated the global “quality of death index,” revealing that wealthier nations dominated the rankings. Brazil ranked 42nd among 83 countries and was the second lowest in South America, with only 0.3% of its population accessing palliative care—highlighting the urgent need for improvement^2^. A subsequent international assessment of the quality of the dying process placed Brazil among the three lowest-performing countries, alongside Paraguay and Lebanon^3^. A cultural stigma in Brazil often associates palliative care exclusively with “end-of-life” or “terminality,” rather than viewing it as an approach aimed at improving quality of life from the diagnosis of a serious illness. This lack of understanding impacts not only the search for these services but also interest and investment in research in the field^4^.

Providing high-quality end-of-life care for patients and their families is crucial, as the circumstances surrounding an individual’s passing significantly affect the bereaved. Early and meaningful discussions about end-of-life care can ease grief complications^5^. To enhance care, reliable tools are needed to assess current practices. Originally developed and validated in the United Kingdom for use in community settings, the Care of the Dying Evaluation (CODE^TM^) questionnaire has demonstrated its importance and validity across diverse international contexts. The tool has been translated into multiple languages, including Spanish, German, Norwegian, Chinese, Dutch, Polish, Persian, Slovenian, Swedish, and Icelandic, and underwent international validation across seven countries for use in hospital settings. Further cultural adaptations for use in Japan and Thailand are currently underway, reinforcing the global applicability and feasibility of cross-cultural validation of the instrument^5,6,7^.

This study aimed to translate, conduct cross-cultural adaptation, and validate CODE^TM^ into Portuguese, while also evaluating care quality in Brazilian hospitals to improve end-of-life care for cancer patients. Additionally, we wanted to undertake an initial assessment of the quality of care using this tool in specific hospitals within Brazil to guide the improvement of the care provided to cancer patients at the end of life.

## METHODS

The study obtained approval from the Research Ethics Committee of Unicamp under opinion number 4.395.657 and from the National Research Ethics Commission under opinion number 2.308.216. All participants signed the Informed Consent Form.

The final study sample in Brazil consisted of 235 bereaved family members. The aim was for each country to collect at least 100 questionnaires, but to conclude the validation in Brazil, we needed at least 220 completed questionnaires.

Recruitment Strategy: The researcher invited family members to participate in the study through an initial phone call, which took place between 6 and 8 weeks after the patient’s death. During the call, the study was explained, and any questions were answered. Participants could choose to complete the questionnaire in person at the Hospital Estadual Sumaré or over the phone. For those who preferred an in-person interview, the Informed Consent Form was signed on-site. For phone interviews, the form was sent by email to be signed and returned as a scanned copy. The validated instrument is the CODE^TM^ questionnaire. It consists of 42 questions divided into sections covering different aspects of care. The answers are classified on nominal and ordinal scales, with answer options ranging from 2 to 5, where scores from 0 to 4 indicate the quality of care (from lowest to highest).

The study consisted of two work packages: WP1 focused on translating, culturally adapting, pre-testing, and validating CODE^TM^, while WP2 involved a post-bereavement survey with next-of-kin of deceased cancer patients. This research was part of a broader international study across hospitals in seven South American and European countries^6^. In Brazil, it was conducted at two public hospitals, Hospital Estadual Sumaré and Hospital de Clínicas, managed by the State University of Campinas. CODE^TM^ consists of six sections, containing 32 main questions (Q) about care and support, such as pain control, communication with the healthcare team, emotional and spiritual support, and circumstances surrounding death. There is an additional section of ten questions about demographic factors and free text space at the end of the questionnaire. The translation followed the European Organisation for Research and Treatment of Cancer (EORTC) protocol, involving forward and back translations, and review by experts to consider Brazil’s cultural context. Two bilingual professionals independently carried out the initial translation^8^.

To ensure appropriate participant selection, ward staff performed a practical assessment of the specified criteria at the time of the patient’s death. This initial screening was followed by further confirmation of eligibility by research personnel during their direct communication with potential participants for study invitation. Throughout this process, all data were collected in strict compliance with applicable data protection laws, guaranteeing participant privacy and data security from the outset.

The use of CODE^TM^ was authorized by the trademark holder, the Palliative Care Unit at the University of Liverpool, UK, through a Material Transfer Agreement. Also, the developer of the original CODE^TM^ tool was a collaborator and coauthor in the present study.

### Cognitive interviewing

Cognitive interviews employed the “think aloud” method, enabling participants to articulate their thoughts and clarify questionnaire terms. The interviews systematically reviewed each section of the CODE^TM^ tool, collecting feedback on question clarity, sensitivity, response options, and recall ability^8,9^. These insights informed refinements to the questionnaire.

WP2—A post-bereavement survey using CODE^TM^ with the next-of-kin to deceased cancer patients.

### Participants

Next-of-kin (≥18 years) of adult cancer patients, whose expected death occurred after ≥72 h of hospitalization with the relative present, were included. The study initially selected 906 bereaved family members for recruitment. Those unable to provide informed consent were excluded. Relatives were invited 6–8 weeks postdeath to complete the CODE^TM^ via face-to-face or phone interview (phone offered due to access/COVID-19 difficulties), after providing prior signed consent (in-person/electronic). Test-retest reliability was checked by phone 2 weeks after the first interview. Questionnaires with insufficient data were excluded.

### Statistical analysis

The construct validity of the CODE^TM^ tool was examined using Confirmatory Factor Analysis, testing two models: Model 1 included all 32 variables, while Model 2 refined the selection for better fit. Goodness-of-fit was assessed through indices such as ratio ꭓ^2^/degrees of freedom, Root Mean Square Error of Approximation, Standardized Root Mean Squared Residual, Comparative Fit Index, Tucker-Lewis Index, and Goodness-of-Fit Index. The Akaike Information Criterion (AIC) determined the best-adjusted model by selecting the lowest AIC value. Internal consistency was evaluated using the Cronbach α coefficient, with satisfactory values set above 0.7. Spearman’s correlation tests assessed item association and total score relationships, considering r>0.3. Test-retest reliability was measured using Kappa and Intra-class Correlation Coefficients, categorizing results as excellent, good, moderate, or poor. A comparative analysis was performed using the Mann-Whitney U test to validate construct consistency and examine differences in perceived care quality between palliative care recipients and others, with a significance threshold of 5% (p<0.05). All statistical analyses were conducted using Predictive Analytics Soft Ware (PASW) Statistics 25.0 software^10^.

The assessment of care using the CODE^TM^ scale presented descriptive data as percentages, reflecting the proportion of participants satisfied with the palliative care received by their family members. Each item was scored on a binary or ordinal scale from 0 to 4, with “0” and “4” representing the lowest and highest quality of care. Satisfaction was defined as scores above “0” for items with two or three response options, and above “2” for items with four or five response options.

## RESULTS

### WP1: Translation and cross-cultural adaptation

Following EORTC procedures, the “CODE^TM^” tool was adapted to enhance its clarity and cultural relevance for a Brazilian context. These adjustments were informed by cognitive interviews conducted with 10 participants. This group included five bereaved family members (one male and four females, aged 25–61 years) and five healthcare professionals (two physiotherapists, a physician, a psychologist, and a care support manager). The study details the practical contributions of the cognitive interviews through Table 1, which records the feedback and general consensus for each question. These direct suggestions from participants resulted in significant changes, such as:

**Table 1:** Summary of feedback about specific questions from cognitive interviews Standard table for feedback questionnaire Care of the Dying Evaluation: Brazilian Cognitive Interviews (question by question).

Comments	Clarity	Recall	Sensitivity	Response options	Consensus
Q2: nursing needs	Adequate the verb to match Q 01	Ok	Ok	Ok	Change the tense of the verb (use “havia” instead of “houve”)
Q3: comfort	Difficult to understand the term “other parts of the room.”	The hospital had no bed available, and the relative remained on the stretcher.	It was not very good to remember this part of the hospitalization.	Ok	Change other parts to room dependencies.
Q4: privacy	Difficult to understand the term “other parts of the room.”	The hospital had no bed available, and the relative remained on the stretcher.	It was not very good to remember this part of the hospitalisation.	Ok	Change other parts to room dependencies.
Q16: decisions	The verb to involve, in Portuguese, is very much associated with feelings, not with making decisions.	Family members say they have become involved psychologically but have not participated in the decisions.	Changing the verb “to involve” to “participate” would be more appropriate.	Ok	Change the verb to participate.
Q17: fluids	It was challenging to understand the expression “infuse fluid into the vein.”	Ok	Ok	Ok	Change to: Hydration.
Q18: discuss about fluids.	The expression “infuse fluid into the vein” was difficult to understand.	Ok	Ok	Ok	Change to: Hydration.

- Language and Terminology: The verb “to involve” in the question about participation in decisions was changed to “to participate,” as family members associated the former with feelings, not with decision-making. The expression “to infuse liquid into the vein” was replaced with “hydration” to facilitate understanding.
- Cultural Relevance and Comfort: The word “toalete” (toilet) was changed to “banheiro” (bathroom). The question about “room dependencies” was adjusted to reflect the reality of hospital overcrowding, where family members recalled the discomfort of accommodating themselves in chairs.
- Demographic Questions: The ethnicity and religion sections were adapted to reflect the most common groups in Brazil.

### WP2-Post-bereavement survey

A total of 235 next-of-kin participated (26% response rate from 906 potential individuals). The respondent sample was largely female (70.6%), White (66.4%), Catholic/Evangelical (89.4%), aged 30–59 years (70.2%), and predominantly sons/daughters (40%) or spouses (26%) of the deceased. Among the deceased patients, cancer (65%) was the main pre-existing condition, with gastrointestinal (42%) and respiratory (26%) types being most common; 48.9% had received a palliative care approach prior to death (Table 2).

**Table 2:** Demographic details of the deceased individuals and participants (n=235).

Deceased Individuals	n (%)
Relationship to deceased patient	
Spouse/partner	61 (26.0)
Son/daughter	94 (40.0)
Brother/sister	32 (13.6)
Son-in-law/daughter-in-law	20 (8.5)
Parent	8 (3.4)
Friend	3 (1.3)
Home care nurse	8 (3.4)
Other	9 (3.8)
Age	
18–19 years	3 (1.3)
20–29 years	17 (7.2)
30–39 years	48 (20.4)
40–49 years	57 (24.3)
50–59 years	60 (25.5)
60–69	37 (15.7)
70–79	13 (5.5)
80–89	0 (0.0)
90 years or more	0 (0.0)
Ethnicity	
White	157 (66.8)
Black	12 (5.1)
Yellow	2 (0.9)
Mixed	64 (27.2)
Gender	
Male	68 (28.9)
Female	167 (71.1)
Religion	
Catholic/evangelical	212 (90.2)
Jewish	1 (0.4)
Spiritualist	22 (9.4)
Patient data	
Pre-existing conditions^*^	
Cancer	186 (65.0)
Heart failure	11 (3.8)
COPD	25 (8.7)
Kidney disease	20 (7.0)
Dementia	1 (0.3)
Motor neuron disease	7 (2.4)
Don´t know	16 (5.6)
Other	20 (7.0)
Age	
18–19 years	2 (0.9)
20–29 years	4 (1.7)
30–39 years	7 (3.0)
40–49 years	16 (6.8)
50–59 years	54 (23.0)
60–69 years	67 (28.5)
70–79 years	60 (25.5)
80–89 years	24 (10.2)
90 years or more	1 (0.4)
Etnicity	
White	169 (71.9)
Black	12 (5.1)
Yellow	2 (0.9)
Mixed	52 (22.1)
Gender	
Male	142 (60.0)
Female	93 (40.0)
Religion	
Catholic/evangelical	214 (91.1)
Jewish	1 (0.4)
Spiritualist	20 (8.5)
Palliative care	
Yes	115 (49)
No	120 (51)
Cancer diagnosis	
Gastrointestinal	99 (42)
Respiratory organs	60 (26)
Gynecological	22 (9)
Brain	11 (5)
Other	43 (18)

*The same patient may have had more than one disease. COPD: chronic obstructive pulmonary disease.

### Care of the Dying Evaluation validation process

#### Confirmatory factor analysis

In Model 1, all 32 CODE^TM^ items were included, but its construct validity was not confirmed due to poor model fit indices. Model 2, refined by excluding variables with low factor loading, confirmed construct validity with four factors, sessions A, B, D, and F and 13 questions. Analysis between the correlation matrix and the factor loadings within Model 2 showed all correlations had values of r>0.37 (p<0.001), demonstrating that each scale represents a single construct (Table 3).

**Table 3:** Analysis of the internal consistency, reproducibility and construct validity of the Care of the Dying Evaluation scale—model 2.

Items by section	Median	1ºQ	3ºQ	Internal consistency		Construct validity	
				α-Cronbach	Total item correlation	Correlation matrix	Factor loading^a^
Section A—Section A: The care received from the nurses and doctors.				**0.68**		**0.25–0.44**	
3. The bed area and surrounding environment were comfortable for him/her.	4	3	4		0.62^**^		0.61**
5. In your opinion, how clean was the ward area that s/he was in?	4	2	4		0.68**		0.46**
6. Did you have confidence and trust in the nurses who were caring for him/her?	4	2	4		0.77**		0.68**
8. The nurses had time to listen and discuss his/her condition with me.	3	3	4		0.75**		0.62**
Section B: The control of pain and other symptoms				**0.65**		**0.39–0.42**	
11. In your view, did the doctors and nurses do enough to help relieve the pain??	4	4	4		0.74**		0.65**
13. In your view, did the doctors and nurses do enough to help relieve the restlessness?	4	3	4		0.76**		0.61**
15. In your view, did the doctors and nurses do enough to help relieve the ‘noisy rattle’ to his/her breathing?	4	4	4		0.75**		0.60**
Section D: The emotional and spiritual support provided by the healthcare team.				**0.8**		**0.35–0.92**	
20. How would you assess the overall level of emotional support given to you by the healthcare team?	3	2	3		0.67**		0.42**
21. Overall, his/her religious or spiritual needs were met by the healthcare team.	3	3	4		0.88**		0.96**
22. Overall, my religious or spiritual needs were met by the healthcare team.	3	3	4		0.91**		0.97**
Section F: Overall impressions				**0.84**		**0.67–0.86**	
30.1 How much of the time was s/he treated with respect and dignity in the last two days of life? (doctors)	4	3	4		0.85**		0.86**
30.2 How much of the time was s/he treated with respect and dignity in the last two days of life? (nurses)	4	3	4		0.93**		0.90**
32. How likely are you to recommend our hospital to friends and family?	4	3	4		0.88**		0.68**

1ºQ: First quartile; 3ºQ: Upper quartile.

**p<0.001;

^a^Factor loading represents the correlation between the factor and the question.

#### Internal consistency

Model 1 showed weak internal consistency, with α-Cronbach values >0.7 only in sections A, B, D, and F, and around 50% of items had total session score correlations below r<0.7. In contrast, Model 2 demonstrated good consistency in sections D and F and moderate consistency in sections A and B. Most item-total score correlations exceeded r>0.7, suggesting the items are interconnected yet perform well as separate scales.

#### Reproducibility

The assessment tool demonstrated excellent test-retest reliability (Kappa>0.94, ICC>0.99). Using CODE^TM^, over 75% of participants reported superior quality palliative care for most items (26 out of 32). However, there was no variation in specific areas as: 58% were satisfied with ward cleanliness, and 47.4% reported being informed about or discussing hydration management near the end of life (Q18). Concerning the dying process (Section E), 50.7% received information about what to expect (Q24), with 37.1% of this subgroup confirming the conversation was helpful (Q25).

Overall, 76.1% reported receiving necessary spiritual and psychological support, a factor associated with receiving palliative care. Significantly, participants whose relatives received palliative care consistently gave higher scores across all CODE^TM^ care sections than those whose relatives did not.

## DISCUSSION

This study provides initial evidence for the validity and reliability of the first Portuguese tool assessing quality of care for dying individuals. It highlights that those supported by a palliative care team perceived higher quality of care than those without such support. The tool underwent a cross-cultural adaptation process, with input from bereaved relatives ensuring clarity and sensitivity in its final version^11^.

Confirmatory factor analysis showed that Model 1 did not confirm construct validity for the six sections of the CODE^TM^ tool. However, Model 2, refined for better fit, validated sections on overall care, symptom management, emotional/spiritual support, and general impressions. Internal consistency was strong in sections D and F and moderate in sections A and B, aligning with previous international validation studies. Test/retest reproducibility in the Brazilian population was excellent (Kappa>0.94). For international use, some items were excluded for statistical purposes, and an abbreviated version of the CODE^TM^ tool was recommended^6^.

The lack of confirmation for Model 1 does not invalidate the use of CODE^TM^ in Brazil. While the original UK model was based on three scales (“Environment,” “Care,” “Communication”), the international validation study—including Brazil—identified a more robust four-factor structure: Overall care, communication and support, trust, respect and dignity, and symptom management. This refined structure, supported by a bifactor model, confirms the instrument’s validity and reliability across countries. Importantly, CODE^TM^ proved sensitive to real differences in quality of care, as shown by higher scores in countries with specialized palliative care units (e.g., Poland). These findings highlight both the cultural adaptability and the robustness of CODE^TM^ as a cross-cultural assessment tool. The moderate consistency in some factors likely reflects true differences in perceptions and experiences rather than a flaw in the instrument itself. The study’s authors acknowledged that these differences might be picking up real variations in the quality of care across different countries and settings.

While a very low Cronbach’s alpha was observed in the “Communication” subscale (α=0.12), the study’s reliance on Confirmatory Factor Analysis (CFA) provides a more robust defense. The CFA results showed that a refined 4-factor model fits the data significantly better than the initial 6-factor model (Root Mean Square Error of Approximation [RMSEA]=0.072 vs. 0.099). This demonstrates that a suitable construct for the instrument was successfully validated, addressing the underlying concern about dimensional structure. This approach is consistent with the international study’s methodology^6^.

Key findings from questionnaire responses demonstrated issues with the hospital environment, for example, relating to cleanliness. This is a particularly important fact, since both hospitals are exclusively public hospitals, where overcrowding and lack of beds are frequent problems. Another relevant fact is that even though two public hospitals suffer from overcrowding and with a budget that is less than ideal, it was demonstrated by this study that 94.8% of the interviewees agreed that there was help available for the personal needs of their family member, that nursing care, such as positioning in bed in a comfortable way, was adequate (94.4%), that there was privacy (86.9%), and that 99% felt safe with care provided by the assistant team^12,13^.

Decisions related to hydration or artificial nutrition are ethically difficult^14,15^. Convictions based on cultural and religious beliefs, embodied in the expression “*do not let an individual die of hunger or thirst*,” lead to the perception that artificial hydration and nutrition should be a rule. It is necessary to have effective and adequate communication with patients and their families^6,16,17,18^.

Brazil’s cultural and religious beliefs can influence the evaluation of the quality of end-of-life care. The discussion around death is often a taboo, influenced by a history of immigration that prioritized life over death. Furthermore, common beliefs, such as the necessity of artificial hydration and nutrition, impose emotional burdens on families and healthcare providers. The study recognizes that spirituality is an indicator of good end-of-life care and must be addressed with respect for the patient’s and family’s cultural and religious values^19^.

Previous studies assessing this aspect reported that although 87% of the participants had access to information about the end of life of their family member, only 63% were informed about what to expect during this phase, such as what symptoms they would witness in the process of death, and reported the paramount importance of this knowledge^20,21,22^.

A key finding demonstrated statistically significant differences (p<0.001) across all CODE^TM^ scales, indicating that relatives reported higher perceived quality of care—including symptom control, communication, and support—when a palliative care team was involved. This underscores the positive contribution of palliative care teams to the overall end-of-life care experience. While the benefits of palliative care models are documented, practical improvements in terminal care remain limited, and tools assessing care quality from the bereaved family’s perspective were previously lacking. Consequently, the CODE^TM^ questionnaire emerges as a valuable instrument to guide and enhance end-of-life care, particularly relevant within the context of Brazil’s recently instituted, 2024, National Palliative Care Policy^23^.

The establishment of Brazil’s 2024 National Palliative Care Policy offers a timely framework for integrating the CODE^TM^ tool into public health services. Demonstrating robust validity and reliability in the Brazilian context, this questionnaire is positioned as a pivotal instrument to guide and enhance end-of-life care. CODE^TM^ can facilitate systematic audits of care quality, providing insights from the perspective of bereaved relatives. Moreover, its findings can inform and refine professional training programs for healthcare providers, addressing critical areas such as symptom management, communication, and emotional support. Ultimately, by yielding concrete quality indicators, the CODE^TM^ tool supports a more comprehensive and patient-centered palliative care approach, fostering continuous improvement and accountability within the new national policy^23^.

This study faced several limitations. It only included relatives of hospitalized cancer patients, highlighting the need for further research in non-cancer populations and different care settings. CODE^TM^ was also validated for home deaths from advanced illnesses, including cancer, cardiac failure, chronic obstructive pulmonary disease (COPD), and renal disease. The tool proved valid, reliable, and user-friendly in this context. Cognitive interviews led to minor adaptations—such as omitting items on ward cleanliness, less relevant at home—highlighting the need for context-specific refinement. Its successful use in a mixed cohort suggests CODE^TM^ is applicable beyond cancer, although further studies in diverse non-cancer populations are warranted^24,25^.

The Covid-19 pandemic posed challenges in administering the questionnaire. Additionally, cultural and religious beliefs, such as the idea that one should not “die of hunger or thirst,” complicated symptom management related to artificial hydration and nutrition at the end of life.

This study is the first translation, cultural adaptation, and validation of CODE^TM^ in Portuguese, addressing a major methodological gap in Brazil. Its contributions are threefold: it enables systematic evaluation and quality improvement of end-of-life care; it incorporates the perspective of bereaved relatives as part of the unit of care; and it provides a validated tool to support future research and inform public policy.

Future research should include multicenter studies across different Brazilian regions to capture regional inequalities, as well as longitudinal designs following bereaved families over time. Comparative studies with other Latin American countries would also be valuable, particularly since similar validation processes are already underway in Argentina and Uruguay. Such efforts will enable regional benchmarking and contribute to a broader understanding of the quality of end-of-life care in diverse contexts.

## CONCLUSION

This study is a pioneer in the translation and validation of the CODE^TM^ tool for the Portuguese of Brazil to evaluate the quality of care offered to cancer patients at the end of life who die in hospitals.

## Data Availability

The datasets generated and/or analyzed during the current study are available from the corresponding author upon reasonable request.
